# A two-dimensional organic–inorganic hybrid compound, poly[(ethylenediamine)tri-μ-oxido-oxidocopper(II)molybdenum(VI)]

**DOI:** 10.1107/S160053680802792X

**Published:** 2008-09-06

**Authors:** Ozgul Gun, Don VanDerveer, Mehtap Emirdag-Eanes

**Affiliations:** a Izmir Institute of Technology, Department of Chemistry, Izmir 35430, Turkey; b Department of Chemistry, Clemson University, Hunter Research Laboratory, Clemson, SC 29634-0973, USA

## Abstract

A new organic–inorganic two-dimensional hybrid compound, [CuMoO_4_(C_2_H_8_N_2_)], has been hydro­thermally synthesized at 443 K. The unit cell contains layers composed of CuN_2_O_4_ octa­hedra and MoO_4_ tetra­hedra. Corner-sharing MoO_4_ and CuN_2_O_4_ polyhedra form CuMoO_4_ bimetallic sites that are joined together through O atoms, forming an edge-sharing Cu_2_Mo_2_O_4_ chain along the *c* axis. The one-dimensional chains are further linked through bridging O atoms that join the Cu and Mo atoms into respective chains along the *b* axis, thus establishing layers in the *bc* plane. The ethyl­enediamine ligand is coordinated to the Cu atom through its two N atoms and is oriented perpendicularly to the two-dimensional –Cu—O—Mo– layers. The average distance between adjacent layers, as calculated by consideration of the closest and furthest distances between two layers, is 8.7 Å. The oxidation states of the Mo and Cu atoms of VI and II, respectively, were confirmed by bond-valence sum calculations.

## Related literature

For related literature on inorganic–organic hybrid materials, see: Gopalakrishnan (1995 ▶); Katsoulis (1998 ▶); Kresge *et al.* (1992 ▶). For related structures containing molybdate(VI) units, see: Cui *et al.* (2005 ▶); Niven *et al.* (1991 ▶). For the thermal behaviour of a related ethyl­enediamine-containing compound, see: Han *et al.* (2005 ▶). For general background, see: Brown & Altermatt (1985 ▶).
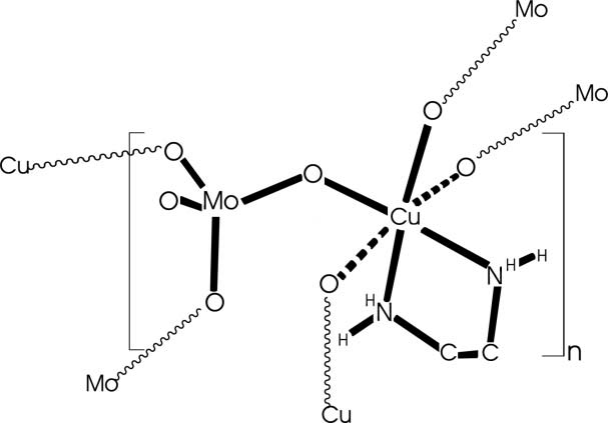


## Experimental

### 

#### Crystal data

[CuMoO_4_(C_2_H_8_N_2_)]
*M* *_r_* = 283.58
Monoclinic, 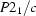

*a* = 9.954 (4) Å
*b* = 9.436 (4) Å
*c* = 7.674 (3) Å
β = 107.734 (18)°
*V* = 686.6 (5) Å^3^
*Z* = 4
Mo *K*α radiation
μ = 4.88 mm^−1^
*T* = 303 (2) K
0.41 × 0.06 × 0.02 mm

#### Data collection

Rigaku Mercury CCD diffractometer
Absorption correction: multi-scan (REQAB; Jacobson, 1998 ▶) *T*_min_ = 0.678, *T*_max_ = 1.000 (expected range = 0.615–0.907)
5616 measured reflections
1209 independent reflections
1098 reflections with *I* > 2σ(*I*)
*R*_int_ = 0.089

#### Refinement

*R*[*F* ^2^ > 2σ(*F* ^2^)] = 0.063
*wR*(*F* ^2^) = 0.111
*S* = 1.10
1209 reflections
91 parameters
H-atom parameters constrained
Δρ_max_ = 0.79 e Å^−3^
Δρ_min_ = −0.92 e Å^−3^

### 

Data collection: *CrystalClear* (Rigaku/MSC, 2001 ▶); cell refinement: *CrystalClear*; data reduction: *CrystalClear*; program(s) used to solve structure: *SHELXTL* (Sheldrick, 2008 ▶); program(s) used to refine structure: *SHELXTL*; molecular graphics: *SHELXTL* software used to prepare material for publication: *SHELXTL*.

## Supplementary Material

Crystal structure: contains datablocks global, I. DOI: 10.1107/S160053680802792X/wm2192sup1.cif

Structure factors: contains datablocks I. DOI: 10.1107/S160053680802792X/wm2192Isup2.hkl

Additional supplementary materials:  crystallographic information; 3D view; checkCIF report

## Figures and Tables

**Table 1:** Selected bond lengths (Å)

Mo1—O2	1.739 (7)
Mo1—O3	1.740 (7)
Mo1—O4	1.789 (7)
Mo1—O1	1.803 (6)
Cu2—O1	1.947 (7)
Cu2—O4^i^	1.951 (7)
Cu2—O1A^ii^	2.574 (7)
Cu2—O3A^iii^	2.460 (7)
Cu2—N2	2.014 (8)
Cu2—N1	2.020 (9)

Symmetry codes: (i) 
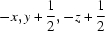
; (ii) 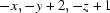; (iii) 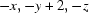.

**Fig. 1.**
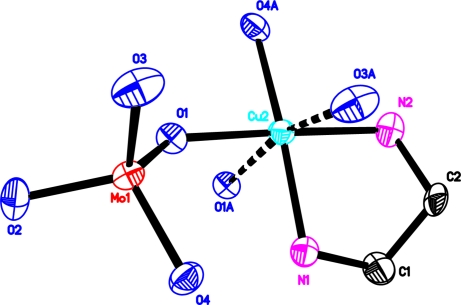
The coordination spheres around Cu and Mo atoms, drawn with displacement ellipsoids at the 50% probability level. H atoms of the en ligand are omitted for clarity. [Symmetry operators to generate equivalent atoms: O1A = -x, 2-y, 1-z; O3A = -x, 2-y, -z O4A = -x, 1/2+y, 1/2-z.]

**Fig. 2.**
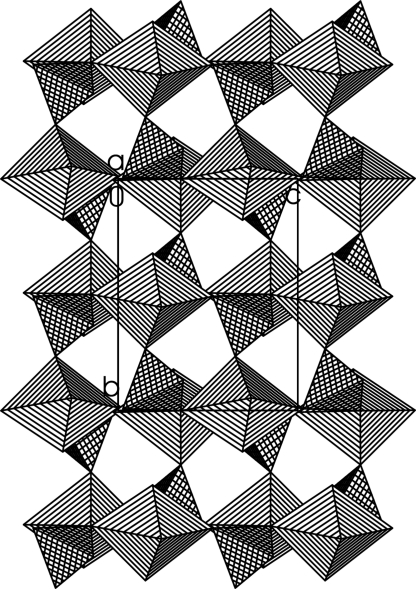
Polyhedra projection of the 1-D chain. CuN2O4 are shown as lined polyhedra, and MoO4 tetrahedra are hatched polyhedra.

**Fig. 3.**
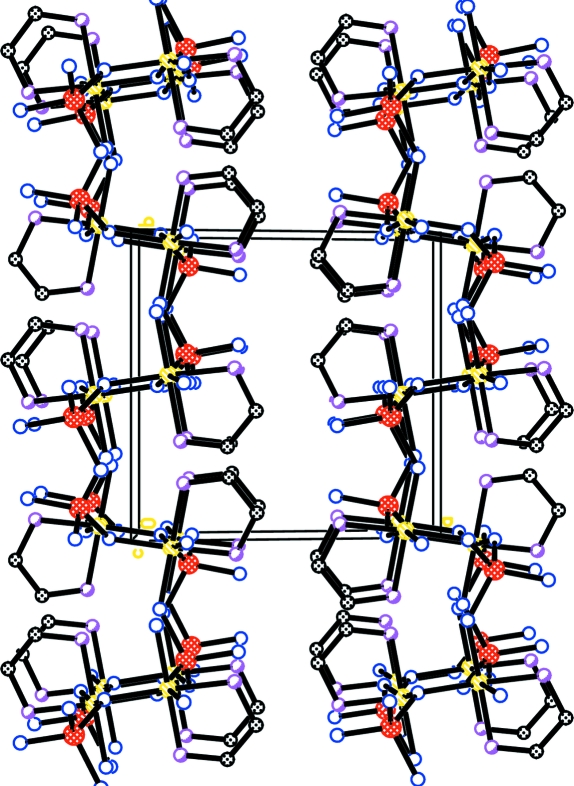
The two-dimensional –Cu—O—Mo- layers. Mo atoms are hatched circles, Cu atoms are lined circles, O atoms are open circles, C atoms are dotted circles and N atoms are shaded circles.

## supplementary crystallographic information

### Comment

The synthesis and characterization of organic inorganic solid state hybrid materials has attracted great attention due to their structural diversity (Kresge *et al.*, 1992) and widely promising potential applications in chemistry, biology and material science (Katsoulis, 1998). Recent studies have shown that hydrothermal synthesis at low temperature and pressure provides a powerful tool for the synthesis of organic inorganic hybrid materials (Gopalakrishnan, 1995).

The unit cell of the title compound, (I), [Cu(en)MoO_4_] (en = ethylenediamine), contains layers composed of distorted CuN_2_O_4_ octahedra and MoO_4_ tetrahedra. The coordination environment of the Cu atom comprises two nitrogen atoms (N1 and N2) from the ethylenediamine ligand with Cu—N distances of 2.020 (9) and 2.014 (8) Å, and four bridging oxygen atoms from four adjacent MoO_4_ tetrahedra with Cu—O distances in the range 1.947 (7) to 2.574 (7) Å (Fig. 1), representing the usual Jahn-Teller distorted coordination.

The Mo atom is coordinated by one terminal oxygen atom (O2) with a distance of 1.739 (7) Å that is indicative of a double bond (Cui *et al.*, 2005) and comparable to other molybdate complexes (Niven *et al.*, 1991). The Mo centre also has two µ_2_ bridging O atoms (O1 and O4), as well as a µ_3_ bridging O atom (O1) with Mo—O bond lengths between 1.739 (7) and 1.803 (6) Å. Corner-sharing MoO_4_ and CuN_2_O_4_ polyhedra form CuMoO_4_ bimetallic sites, and the CuMoO_4_ groups are joined together through the O1 atoms forming an edge-sharing Cu_2_Mo_2_O_4_ chain along the *c* axis (Fig. 2). The one-dimensional chains are linked through bridging O4 atoms, that bind the Cu and Mo atoms in the respective chains along the *b* axis, to establish layers in the *bc*-plane. The en ligand is coordinated to the copper atom through its two nitrogen atoms and is oriented perpendicularly to the two-dimensional –Cu—O—Mo- layers (Fig. 3). The average distance between two layers, as calculated under consideration of the closest and furthest distances between two adjacent layers, is 8.7 Å.

The +VI oxidation state of the Mo atoms and the +II oxidation state of the Cu atoms were confirmed by bond valence sum calculations (Brown & Altermatt, 1985). The calculated bond valence values for the Mo and Cu atoms are 5.86 and 1.94 Å, respectively.

The thermal behaviour of the title compound was studied in the range 298–923 K under nitrogen atmosphere, demonstrating that the compound is stable up to 468 K. The TG curve exhibits two steps of weight loss. While the first weight loss is 14.61% in the temperature range 468 to 513 K, the second is 6.78% between 513 to 723 K. The total weight loss from 468 to 723 K thus becomes 21.39%, corresponding to the removal of an ethylenediamine group in agreement with the calculated value of 21.15%. These results are comparable to the thermal behaviour of the related compound [Cd(en)_3_]MoO_4_ (Han *et al.*, 2005).

### Experimental

Compound (I) was synthesized *via* a hydrothermal reaction procedure. The following reagents were used as obtained; Na_2_MoO_4_^.^2H_2_O (Carlo Erba, 99.5%), CuCl_2_^.^2H_2_O (Sigma, 99.6%), NaCl (Merck, >99%), and en (Merck, >99%). A mixture of Na_2_MoO_4_^.^2H_2_O (0.2420 g, 1 mmol), CuCl_2_^.^2H_2_O (0.3410 g, 2 mmol), NaCl (0.0585 g, 1 mmol), en (0.2 ml, 3 mmol) and water (9 ml, 500 mmol) in a molar ratio of 1:2:1:3:500, was loaded into a 23 ml Teflon-lined stainless steel autoclave and heated at 443 K for 72 h. After slow cooling to room temperature, blue crystals with columnar habit of the title compound were recovered in a 90% yield by suction filtration and washed with water and acetone.

### Refinement

H atoms were placed in idealised positions and refined in the riding model approximation with a C—H distance of 0.96 Å and U_iso_(H) = 1.2× U_eq_(C), and with a N—H distance of 0.90 Å and U_iso_(H) = 1.2× U_eq_(N), respectively.

### Crystal data

**Table:**

[CuMoO_4_(C_2_H_8_N_2_)]	*F*(000) = 548
*M**_r_* = 283.58	*D*_x_ = 2.743 Mg m^−^^3^
Monoclinic, *P*2_1_/*c*	Mo *K*α radiation, λ = 0.71073 Å
Hall symbol: -P 2ybc	Cell parameters from 4208 reflections
*a* = 9.954 (4) Å	θ = 2.2–26.0°
*b* = 9.436 (4) Å	µ = 4.88 mm^−^^1^
*c* = 7.674 (3) Å	*T* = 303 K
β = 107.734 (18)°	Column, blue
*V* = 686.6 (5) Å^3^	0.41 × 0.06 × 0.02 mm
*Z* = 4	

### Data collection

**Table:**

Rigaku Mercury CCD diffractometer	1209 independent reflections
Radiation source: Sealed Tube	1098 reflections with *I* > 2σ(*I*)
Graphite Monochromator	*R*_int_ = 0.089
Detector resolution: 14.6199 pixels mm^-1^	θ_max_ = 25.0°, θ_min_ = 3.1°
ω–scans	*h* = −11→11
Absorption correction: multi-scan (REQAB; Jacobson, 1998)	*k* = −11→11
*T*_min_ = 0.678, *T*_max_ = 1.000	*l* = −8→9
5616 measured reflections	

### Refinement

**Table:**

Refinement on *F*^2^	Primary atom site location: structure-invariant direct methods
Least-squares matrix: full	Secondary atom site location: difference Fourier map
*R*[*F*^2^ > 2σ(*F*^2^)] = 0.063	Hydrogen site location: inferred from neighbouring sites
*wR*(*F*^2^) = 0.111	H-atom parameters constrained
*S* = 1.10	*w* = 1/[σ^2^(*F*_o_^2^) + (0.0154*P*)^2^ + 14.9224*P*] where *P* = (*F*_o_^2^ + 2*F*_c_^2^)/3
1209 reflections	(Δ/σ)_max_ = 0.001
91 parameters	Δρ_max_ = 0.79 e Å^−^^3^
0 restraints	Δρ_min_ = −0.92 e Å^−^^3^

### Special details

**Table:**

Geometry. All e.s.d.'s (except the e.s.d. in the dihedral angle between two l.s. planes) are estimated using the full covariance matrix. The cell e.s.d.'s are taken into account individually in the estimation of e.s.d.'s in distances, angles and torsion angles; correlations between e.s.d.'s in cell parameters are only used when they are defined by crystal symmetry. An approximate (isotropic) treatment of cell e.s.d.'s is used for estimating e.s.d.'s involving l.s. planes.
Refinement. Refinement of *F*^2^ against ALL reflections. The weighted *R*-factor *wR* and goodness of fit *S* are based on *F*^2^, conventional *R*-factors *R* are based on *F*, with *F* set to zero for negative *F*^2^. The threshold expression of *F*^2^ > σ(*F*^2^) is used only for calculating *R*-factors(gt) *etc*. and is not relevant to the choice of reflections for refinement. *R*-factors based on *F*^2^ are statistically about twice as large as those based on *F*, and *R*- factors based on ALL data will be even larger.

### Fractional atomic coordinates and isotropic or equivalent isotropic displacement parameters (Å^2^)

**Table:**

	*x*	*y*	*z*	*U*_iso_*/*U*_eq_	
Mo1	0.16945 (9)	0.89946 (9)	0.21937 (11)	0.0186 (3)	
Cu2	−0.12230 (13)	1.03150 (13)	0.30706 (17)	0.0205 (4)	
N1	−0.1618 (9)	0.8217 (9)	0.3090 (11)	0.0226 (19)	
H1A	−0.1479	0.7798	0.2106	0.027*	
H1B	−0.1035	0.7815	0.4103	0.027*	
O1	0.0784 (7)	1.0015 (7)	0.3471 (9)	0.0212 (15)	
N2	−0.3317 (9)	1.0540 (10)	0.2586 (11)	0.025 (2)	
H2A	−0.3505	1.0774	0.3625	0.030*	
H2B	−0.3651	1.1230	0.1758	0.030*	
O2	0.3401 (7)	0.8692 (8)	0.3611 (10)	0.0299 (18)	
O3	0.1771 (9)	0.9921 (8)	0.0266 (10)	0.036 (2)	
O4	0.0827 (7)	0.7333 (7)	0.1511 (10)	0.0258 (16)	
C2	−0.3985 (10)	0.9178 (11)	0.1886 (14)	0.024 (2)	
H2C	−0.4021	0.9054	0.0630	0.029*	
H2D	−0.4930	0.9145	0.1962	0.029*	
C1	−0.3096 (11)	0.8039 (12)	0.3058 (16)	0.031 (3)	
H1C	−0.3159	0.8100	0.4279	0.038*	
H1D	−0.3433	0.7123	0.2573	0.0387*	

### Atomic displacement parameters (Å^2^)

**Table:**

	*U*^11^	*U*^22^	*U*^33^	*U*^12^	*U*^13^	*U*^23^
Mo1	0.0228 (5)	0.0186 (4)	0.0169 (4)	−0.0027 (4)	0.0104 (3)	−0.0023 (4)
Cu2	0.0204 (7)	0.0178 (6)	0.0233 (7)	0.0005 (5)	0.0074 (5)	0.0011 (5)
N1	0.022 (5)	0.027 (5)	0.019 (4)	−0.001 (4)	0.007 (4)	0.003 (4)
O1	0.025 (4)	0.022 (4)	0.018 (4)	0.003 (3)	0.009 (3)	−0.004 (3)
N2	0.025 (5)	0.036 (5)	0.015 (4)	−0.003 (4)	0.008 (4)	−0.004 (4)
O2	0.018 (4)	0.037 (5)	0.033 (4)	−0.001 (3)	0.006 (3)	−0.011 (4)
O3	0.055 (5)	0.035 (5)	0.023 (4)	−0.012 (4)	0.022 (4)	−0.003 (4)
O4	0.026 (4)	0.022 (4)	0.026 (4)	−0.006 (3)	0.005 (3)	−0.003 (3)
C2	0.012 (5)	0.033 (6)	0.030 (6)	−0.005 (4)	0.008 (4)	0.000 (5)
C1	0.026 (6)	0.036 (7)	0.034 (6)	0.001 (5)	0.014 (5)	0.013 (5)

### Geometric parameters (Å, °)

**Table:**

Mo1—O2	1.739 (7)	N1—H1A	0.9000
Mo1—O3	1.740 (7)	N1—H1B	0.9000
Mo1—O4	1.789 (7)	N2—C2	1.471 (13)
Mo1—O1	1.803 (6)	N2—H2A	0.9000
Cu2—O1	1.947 (7)	N2—H2B	0.9000
Cu2—O4^i^	1.951 (7)	O4—Cu2^iv^	1.951 (7)
Cu2—O1A^ii^	2.574 (7)	C2—C1	1.505 (15)
Cu2—O3A^iii^	2.460 (7)	C2—H2C	0.9600
Cu2—N2	2.014 (8)	C2—H2D	0.9600
Cu2—N1	2.020 (9)	C1—H1C	0.9600
N1—C1	1.473 (13)	C1—H1D	0.9600
			
O2—Mo1—O3	109.1 (4)	C2—N2—Cu2	107.6 (6)
O2—Mo1—O4	109.4 (3)	C2—N2—H2A	110.2
O3—Mo1—O4	109.5 (3)	Cu2—N2—H2A	110.2
O2—Mo1—O1	107.8 (3)	C2—N2—H2B	110.2
O3—Mo1—O1	110.7 (3)	Cu2—N2—H2B	110.2
O4—Mo1—O1	110.4 (3)	H2A—N2—H2B	108.5
O1—Cu2—O4^i^	88.3 (3)	Mo1—O4—Cu2^iv^	138.7 (4)
O1—Cu2—N2	177.3 (3)	N2—C2—C1	106.7 (9)
O4^i^—Cu2—N2	94.2 (3)	N2—C2—H2C	110.4
O1—Cu2—N1	92.8 (3)	C1—C2—H2C	110.4
O4^i^—Cu2—N1	170.2 (3)	N2—C2—H2D	110.4
N2—Cu2—N1	84.9 (3)	C1—C2—H2D	110.4
C1—N1—Cu2	107.9 (6)	H2C—C2—H2D	108.6
C1—N1—H1A	110.1	N1—C1—C2	109.3 (8)
Cu2—N1—H1A	110.1	N1—C1—H1C	109.8
C1—N1—H1B	110.1	C2—C1—H1C	109.8
Cu2—N1—H1B	110.1	N1—C1—H1D	109.8
H1A—N1—H1B	108.4	C2—C1—H1D	109.8
Mo1—O1—Cu2	130.8 (4)	H1C—C1—H1D	108.3
			
O1—Cu2—N1—C1	172.4 (6)	O4^i^—Cu2—N2—C2	170.1 (6)
O4^i^—Cu2—N1—C1	76 (2)	N1—Cu2—N2—C2	−19.7 (6)
N2—Cu2—N1—C1	−9.1 (7)	O2—Mo1—O4—Cu2^iv^	1.6 (7)
O2—Mo1—O1—Cu2	−161.3 (5)	O3—Mo1—O4—Cu2^iv^	121.0 (6)
O3—Mo1—O1—Cu2	79.4 (5)	O1—Mo1—O4—Cu2^iv^	−116.8 (6)
O4—Mo1—O1—Cu2	−42.0 (6)	Cu2—N2—C2—C1	43.7 (9)
O4^i^—Cu2—O1—Mo1	−133.4 (5)	Cu2—N1—C1—C2	36.0 (10)
N1—Cu2—O1—Mo1	56.3 (5)	N2—C2—C1—N1	−53.4 (11)
O1—Cu2—N2—C2	14 (7)		

Symmetry codes: (i) −*x*, *y*+1/2, −*z*+1/2; (ii) −*x*, −*y*+2, −*z*+1; (iii) −*x*, −*y*+2, −*z*; (iv) −*x*, *y*−1/2, −*z*+1/2.
